# CT pulmonary arteriogram diagnosis of macroscopic fat embolism to the lung

**DOI:** 10.1016/j.radcr.2024.02.062

**Published:** 2024-03-05

**Authors:** Robert Murphy, Róisín A. Murray, Sven O'hEireamhoin, John G. Murray

**Affiliations:** a Department of Medicine, Mater Misericordiae University Hospital, Eccles Street, Dublin 7, Dublin, Ireland; b University College Dublin, Dublin 4, Dublin, Ireland; c Department of Radiology, Mater Misericordiae University Hospital, Eccles Street, Dublin 7, Dublin, Ireland; d Present Address: Department of Nursing, University Hospital Galway, Galway city, Ireland; e Department of Orthopaedic Surgery, Mater Misericordiae University Hospital, Eccles Street, Dublin 7, Dublin, Ireland

**Keywords:** CT pulmonary arteriogram, Pulmonary embolism, Pulmonary fat embolism

## Abstract

Pulmonary fat embolism (PFE) is a recognised complication of long bone fractures. The majority of cases represent microscopic embolism and are not detectable at CT pulmonary arteriography (CTPA). CT can be used to detect macroscopic fat based on Hounsfield attenuation. This case describes a case of macroscopic fat embolism to the pulmonary arteries which was confidently diagnosed at CTPA. Distinction from thromboembolism is important as treatment is supportive and may avoid risks of anticoagulation.

## Introduction

CT pulmonary arteriography (CTPA) is the optimal modality for diagnosis of pulmonary embolism (PE). The majority of cases are due to thromboembolism. Other causes include air embolism, cement embolism, foreign body embolism, septic embolism, tumor embolism, hydatid embolism and fat embolism [1], [2], [3], [4], [5], [6], [7], [8]. The majority of cases of fat embolism are due to microscopic fat post long bone fractures or orthopaedic surgery with implants. Microscopic fat embolism does not result in a filling defect in the pulmonary arteries and diagnosis cannot be made on CTPA alone [1]. Macroscopic pulmonary fat embolism is a rare manifestation of fat embolism where macroscopic fat is present within the pulmonary arteries [1], [2], [3], [4], [5]. CT measurement of the Hounsfield attenuation of the pulmonary artery filling defects allowed confident diagnosis of macroscopic fat embolism in this case.

## Case presentation and investigations

A 78-year-old post operative patient presented with increasing shortness of breath and left sided posterior knee pain post recent left hip girdlestone arthroplasty surgery. This was performed for chronic left prosthetic hip joint infection that had failed to resolve following multiple surgical revisions, washouts, and prolonged courses of intravenous antibiotics. Girdlestone surgery involved removal of hip prosthesis and adjacent infected bone. Past medical history included ischaemic heart disease, heart failure, paroxysmal atrial fibrillation, COPD, and peripheral vascular disease.

Patient had no chest pain or haemoptysis. Review of systems was non-contributory. Physical examination showed a heart rate: 67 bpm, blood pressure 157/90 mmHg, respiratory rate 15/min, SpO2 90% on room air, GCS 15/15, Temp 36.7C. Patient was alert, oriented and interactive. Bilateral pitting oedema was present to the knees, more pronounced on the left. Cardiorespiratory examination was otherwise normal. There was no petechial rash.

Initially investigations were significant for NT-ProBNP: 8703 ng/L (<300), high sensitivity troponin: 9 ng/L (< 5) and CRP 26 (< 5). Full blood count, renal function, and liver function tests were normal. Arterial blood gas on room air demonstrated hypoxaemia with P02: 9.57 kPa. PCO2 5.58, Bicarb 26, Lactate 1.8. Differential diagnosis at time of admission including decompensated heart failure with pulmonary oedema and pulmonary embolism in the setting of recent surgery and possible left lower limb DVT. Patient was commenced on therapeutic anticoagulation (Enoxaparin 1 mg/kg twice daily) empirically for likely deep vein thrombosis (DVT) and PE and referred for chest radiograph, Duplex ultrasound left leg and CTPA.

**Fig. 1:**
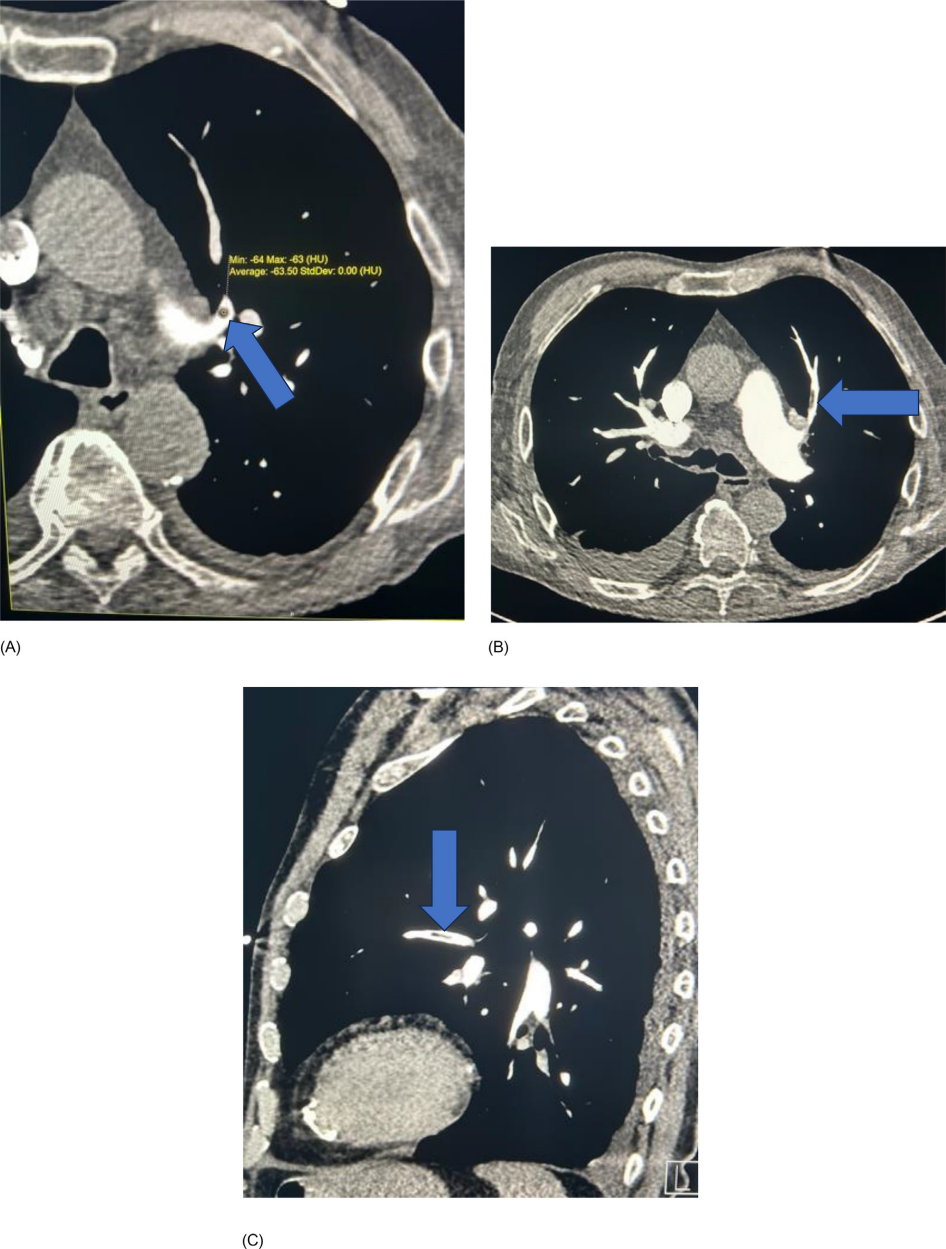
CTPA study. (A) axial magnified image of left lung shows filling defect in left upper lobe pulmonary artery (arrow) with a mean HU of -63.5 indicating fat. (B) axial and (C) sagittal images show further fatty emboli (arrows) to anterior segmental artery upper lobe left lung. Note small dependent bibasal pleural effusions due to heart failure.

**Fig. 2:**
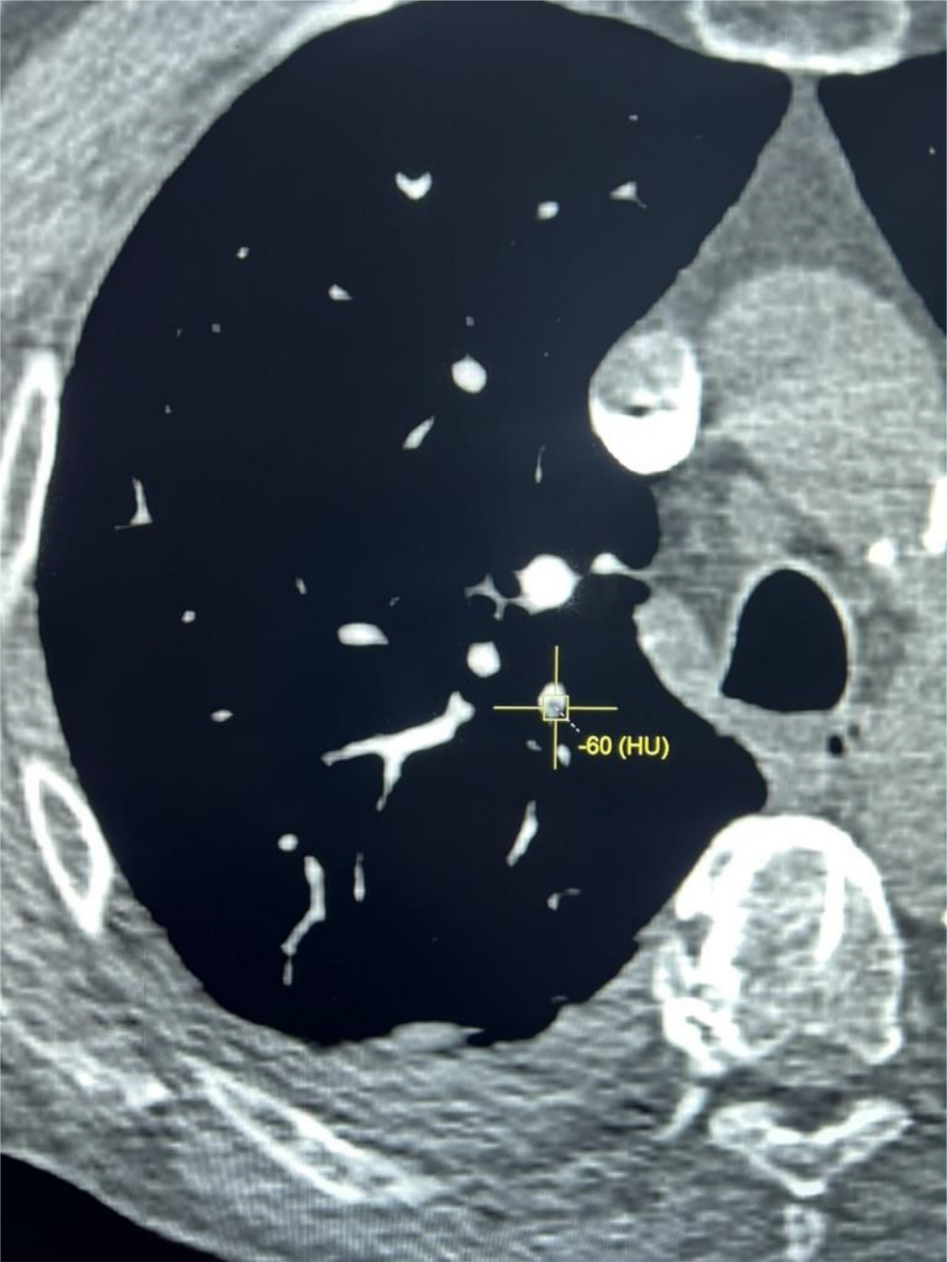
Axial magnified image of right lung shows filling defect in subsegmental branch of upper lobe pulmonary artery (cursor) with a mean HU of -60 indicating fat.

Chest radiograph showed bilateral small pleural effusions and diffusely prominent interstitial markings compatible with pulmonary oedema. US Doppler of the left lower limb demonstrated a nonocclusive chronic thrombus in the left proximal femoral vein measuring approximately 1.5 cm in length, as well as an acute thrombus in the short saphenous vein, beginning at the popliteal junction, and extending down the posterior calf measuring at least 7.5 cm. CTPA exam was performed on Somatom Definition AS+ scanner (Siemens, Erhlangen, Germany). About 60 mLs of Iomeron 350 (Iomeprol, Bracco, UK) followed by 30 cc saline flush was injected via an 18G cannula in right antecubital vein at 5 mLs per second during a single inspiratory breath-hold. Images were reconstructed at 1 mm section thickness and reviewed on PACS workstation (Change Healthcare, Tennessee USA). CTPA exam showed filling defects within the segmental branches of the left upper lobe pulmonary arteries and a subsegmental artery in the right upper lobe. These were of fatty attenuation with a mean CT number of -63.5 Hounsfield units (HU) in the left upper lobe and -60 Hounsfield units in right upper lobe, compatible with fat emboli (Fig. 1, Fig. 2).

Patient was treated with diuresis and supportive measures including nasal oxygen. Haematology team advised Edoxaban 30 mg orally once daily for 3 months for provoked venous thromboembolism. Symptoms improved over hospital stay and patient was discharged 5 weeks later to an off-site rehabilitation hospital.

## Discussion

Fat embolism results from disruption of intramedullary fat following long bone fractures or orthopaedic surgery. Microscopic fat embolism can be detected in the blood and urine of almost all patients with long bone fractures, but only a minority of these develop clinical symptoms [1]. Extensive fat embolism results in fat embolism syndrome which is a clinical diagnosis based on hypoxia, confusion, and a petechial rash, and can progress to adult respiratory distress syndrome (ARDS). Pathophysiology appears to relate to a combination of mechanical obstruction due to fat particles obstructing capillaries with ventilation perfusion mismatch and fatty acid induced inflammatory damage to alveoli with ARDS [1].

Diagnosis of pulmonary fat embolism by CT visualization of macroscopic fat in the pulmonary vasculature is rare [1], [2], [3], [4], [5], [6], [7], [8]. Pulmonary fat embolism was confidently diagnosed in this case due to CTPA demonstration of fat-attenuation filling defects (-63.5 HU and -60 HU) in the pulmonary arteries. Fat typically has an attenuation value of -50 HU to -150 HU, and can be distinguished from both acute and chronic pulmonary thromboembolism which have a mean attenuation value of 33 HU (95% confidence intervals 26, 41 HU) and 87 HU (95% confidence intervals 66, 107 HU) respectively [9]. Certain tumors may contain fat and could potentially metastasize to the lung including renal cell carcinoma, hepatocellular carcinoma, and liposarcoma. Patient had no history of primary tumor, and metastatic disease would typically present as pulmonary nodules and not as a long intraluminal defects as seen in this case (Fig. 1).

Chest radiograph is generally the first imaging test in patients with respiratory distress but findings of fat embolism are nonspecific and indistinguishable from pulmonary oedema, infection or aspiration. CT lung parenchymal findings in fat embolism are also generally nonspecific and include patchy ground glass opacities with consolidation and small centrilobular nodules [1], [2], [3], [4], [5], [6], [7], [8]. CTPA is the test of choice to diagnose pulmonary embolism and in contrast to ventilation perfusion scintigraphy allows direct visualization of embolic material permitting distinction of thromboembolism and fat embolism. In the above case the patient had dual pathology namely a DVT and pulmonary fat embolism and as a result was treated with anticoagulants. However in cases of isolated fat embolism, routine use of heparin is not recommended due to lack of evidence, risk of hamorrhage and the potential, theoretical risk of exacerbation due to production of free fatty acids [6]. Diagnosis of PFE over thrombotic pulmonary embolism may avoid the risks of prolonged treatment with anti-coagulation. This is particularly important in postoperative patients and patients with severe trauma due to the risk of hemorrhage as a complication of treatment. Treatment of isolated fat embolism is predominantly supportive with the majority of patients recovering in 1-2 weeks [1].

In conclusion, Radiologists who interpret CTPA studies of patients with recent severe trauma and long bone injuries or of patients following orthopedic instrumentation, should consider fat embolism as a differential when pulmonary artery filling defects are seen on imaging. Macroscopic pulmonary fat embolism, though rare, can be confidently distinguished from thromboembolism based on its negative Hounsfield attenuation number. This can have important implications for patient management.

## Patient consent

Written informed consent was obtained from the patient.
